# Long-term effects of combined brilliant blue G and xenon light induced retinal toxicity following macular hole repair surgery

**DOI:** 10.1186/s12886-023-02811-w

**Published:** 2023-02-09

**Authors:** Ramesh Venkatesh, Rubble Mangla, Shama Sharief, Jay Chhablani

**Affiliations:** 1 Narayana Nethralaya, Dept. of Retina and Vitreous, #121/C, Chord Road, 1st R block Rajaji Nagar, 560010 Bangalore, India; 2 Medical Retina and Vitreoretinal Surgery, University of Pittsburgh School of Medicine, 203 Lothrop Street, Suite 800, 15213 Pittsburg, PA USA

**Keywords:** Macular hole, Brilliant blue G, Retinal toxicity, Outcomes, Long-term

## Abstract

**Background:**

The purpose of this study was to look at the long-term effects of retinal phototoxicity after macular hole repair surgery using xenon endolight illumination and Brilliant blue G (BBG) dye.

**Case presentation:**

An elderly man in his late seventies underwent para plana vitrectomy with BBG dye to repair an idiopathic full-thickness macular hole (MH) in his right eye. Prior to macular hole surgery, his visual acuity in the right eye was 6/60, N24 at the time of presentation. The MH closed with type 1 closure immediately after surgery, but there was extensive damage to the outer retinal layers and retinal pigment epithelium (RPE) at the macula, resulting in a reduction in visual acuity to 2/60. We presumed that the combination of BBG and xenon light, is the probable reason of retinotoxicity in the current patient. There was a progressive increase in the area of retinal and RPE layer damage and choroidal thinning over a 4-year period.

**Conclusion:**

Due to combined BBG-induced dye and endoilluminator toxicity, a rare case of continuously progressing RPE layer damage with choroidal thinning over a long follow-up interval was described. Such long-term effects of BBG and endolight induced retinotoxicity have not been reported in the literature, to the best of our knowledge.

## Background

A number of pre-, intra- and post-operative factors help to determine the final visual acuity following pars plana vitrectomy and brilliant blue G (BBG)-assisted internal limiting membrane (ILM) peeling for macular hole repair surgery [1, 2]. The postoperative vision depends upon the continuity of the external limiting membrane and inner segment – outer segment layers and the health of the retinal pigment epithelium (RPE) at the fovea. There are a few reports in literature demonstrating the combined toxic effects of BBG and endoilluminator light causing foveal thinning and damage to the outer retinal layers and RPE, thereby causing decreased vision [3–5]. The cases described in these reports demonstrated the short-term (follow-up duration up to 2 months) effects of combined BBG and endolight toxicity on the retina.

The current case report demonstrates the progressive changes in the outer retina, RPE and choroidal layers following the use of BBG over a 4-year follow-up duration after macular hole repair surgery.

## Case presentation

**Fig. 1:**
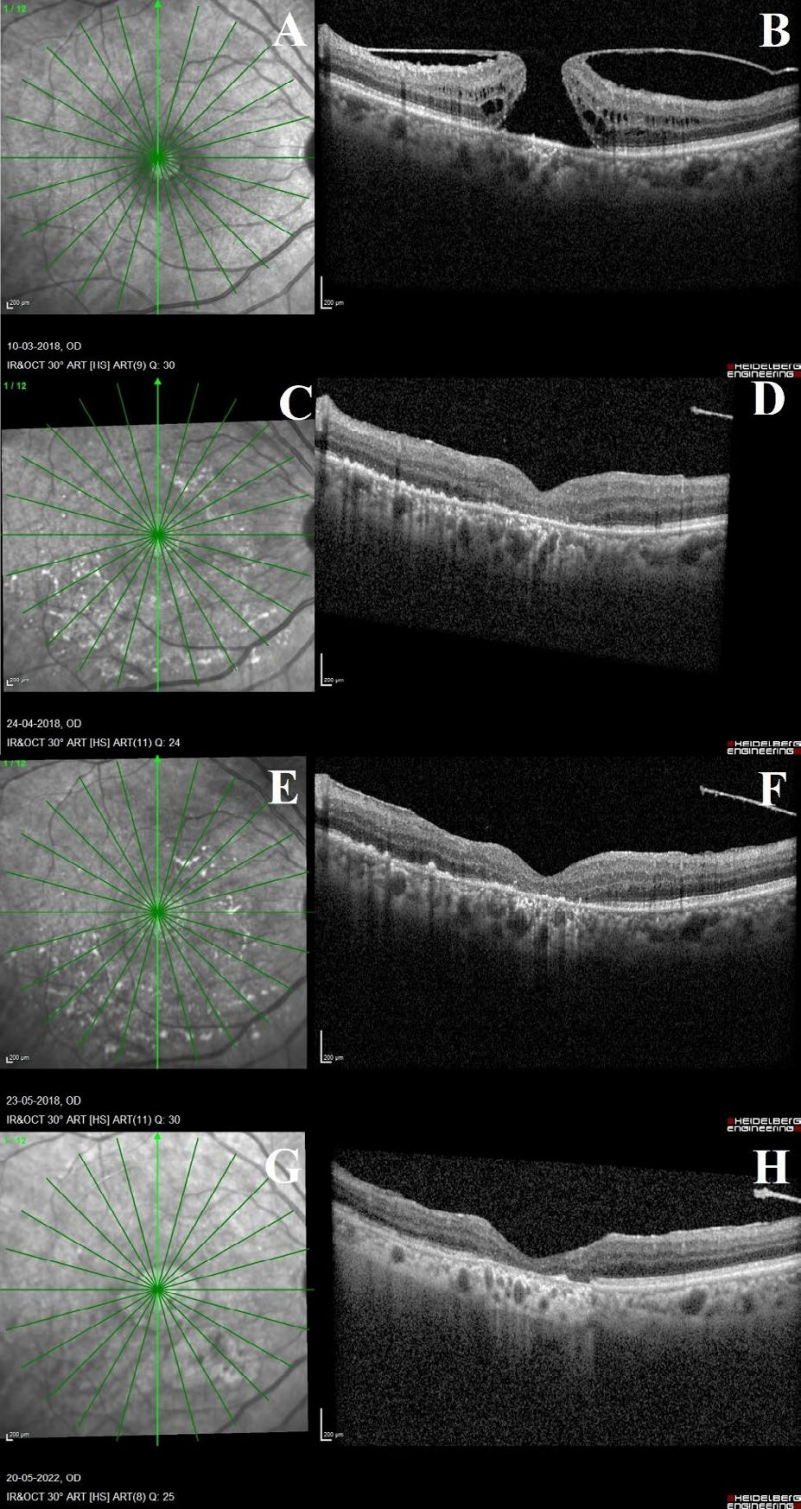
**Sequential pre- and post-operative optical coherence tomography (OCT) and enface infrared (IR) images of the macula in a patient with idiopathic full-thickness macular hole**: Figure 1 A, B: Pre-operative IR and OCT image of the macula showing the full-thickness macular hole with posterior hyaloid attached to its margins and presence of intraretinal fluid filled spaces. The retinal pigment epithelium (RPE) appears normal with normal reflectivity of the underlying choroidal layers. Figure 1 C, D: 2-week post-operative scans showing speckled hyperreflective dots at the macula on the IR image. On the OCT, the macular hole is closed with normal foveal contour with damaged outer retinal layers. RPE changes are identified on the IR image and OCT scan. Figure 1E, F: 6-week post-operative scans show findings similar to that observed on the 2-week post-operative visit. The hyperreflective dots on the IR image have reduced. A type 1 macular hole closure with normal foveal contour with foveal thinning and damaged outer retinal layers and RPE is identified on the OCT scan. Figure 1G, H: 4-year after the macular hole repair surgery, the hyperreflective dots on the IR image have disappeared with two large patches of RPE atrophy identified on the macula; one at the fovea and another inferonasal to the fovea. The OCT scan shows a closed macular hole with complete loss of the RPE layer and associated increased visibility of the underlying choroidal layers

A 77-year-old male with a 15-year duration of type 2 diabetes mellitus was referred from the cataract department to the retina clinic for further management of a full-thickness macular hole in the right eye. In the past six months, he complained of decreased vision in the right eye. There was no history of ocular trauma in the past. On ocular examination, his right and left eye visual acuity were 6/60, N24 and 6/9, N6 respectively. In the right and left eyes, nuclear cataracts of grade 3 and grade 2 severity (Lens Opacities Classification System III) were observed, respectively. The examination of the right eye’s dilated retina revealed a circular, full-thickness retinal defect at the fovea and a taut posterior hyaloid membrane. In the right eye, a mild stage of non-proliferative diabetic retinopathy was observed. The fundus of the left eye was normal. A full-thickness idiopathic macular hole was diagnosed, and an optical coherence tomography (OCT) scan was advised. The OCT scan of the right eye obtained using the Spectralis (Heidelberg Engineering, Germany) and passing through the center of the fovea revealed a full-thickness macular hole with the posterior hyaloid attached to its margins. Inner retinal fluid-filled cystoid spaces were observed. On the vertical radial line scan passing through the center of the fovea, the inner diameter, outer basal diameter, and height of the macular hole were 615 m, 1885 m, and 534 m, respectively. Normal reflectivity was observed in the RPE layer (Fig. 1A-B). The patient underwent a combined phacovitrectomy and macular hole repair 15 days after surgery was suggested. After cataract removal and intraocular lens implantation (Aurovue EV Preloaded, Aurolab, India), a 25-gauge microincision vitrectomy system was performed using the Constellation® (Alcon, USA) vitrectomy machine on the patient’s right eye. Following removal of the core vitreous, posterior vitreous detachment was induced with the vitrectomy cutter assisted by triamcinolone acetonide staining of the posterior cortical vitreous. The flute needle was used for passively performing fluid-air exchange. BBG dye (OcuBlue Plus 0.05%w/v, Aurolab, India) was injected using a 1-cc tuberculin syringe fitted with a flute needle. The dye was slowly injected from the mid vitreous cavity onto the retina in order to cover a larger portion of the posterior pole fundus. At this time, the illuminations of the microscope and endolight were turned off. After a 2-minute incubation period, the BBG dye was removed passively from the vitreous cavity with the aid of a flute needle, and the saline infusion was restarted gradually so as to avoid jet-stream-related retinal injury. With the ILM forceps (GRIESHABER REVOLUTION® DSP ILM FORCEPS) and endoilluminator light intensity of 110%, conventional circular peeling of the ILM was attempted, beginning from the inferonasal margin of the optic disc and extending between the superior and inferior retinal arcades vertically and from the temporal margin of the optic disc to approximately 3-disc diameters temporal to the fovea horizontally. The ILM peeling was ultimately completed after it was stained twice with BBG in air. The duration of the entire ILM peeling was extended due to the inadequate staining of the ILM and subsequently piece-meal ILM removal, bringing the total duration of the surgery to 87 min. During the surgery, no intraoperative complications were observed. After fluid-air exchange with a flute needle, 20% sulfur hexafluoride (SF6) gas was injected as endotamponade, and a 7-day strict prone position was advised. One week following surgery, the patient’s visual acuity documented showed a decline in visual acuity to 2/60, N36, and an OCT scan revealed a closed macular hole with a preserved foveal contour and damaged outer retina and RPE layers. The symptoms were indicative of acute retinal toxicity caused by the combination of BBG and xenon endolight. At the 2-week and 6-week follow-up appointments, the clinical and OCT characteristics persisted (Fig. 1C-F).

**Fig. 2:**
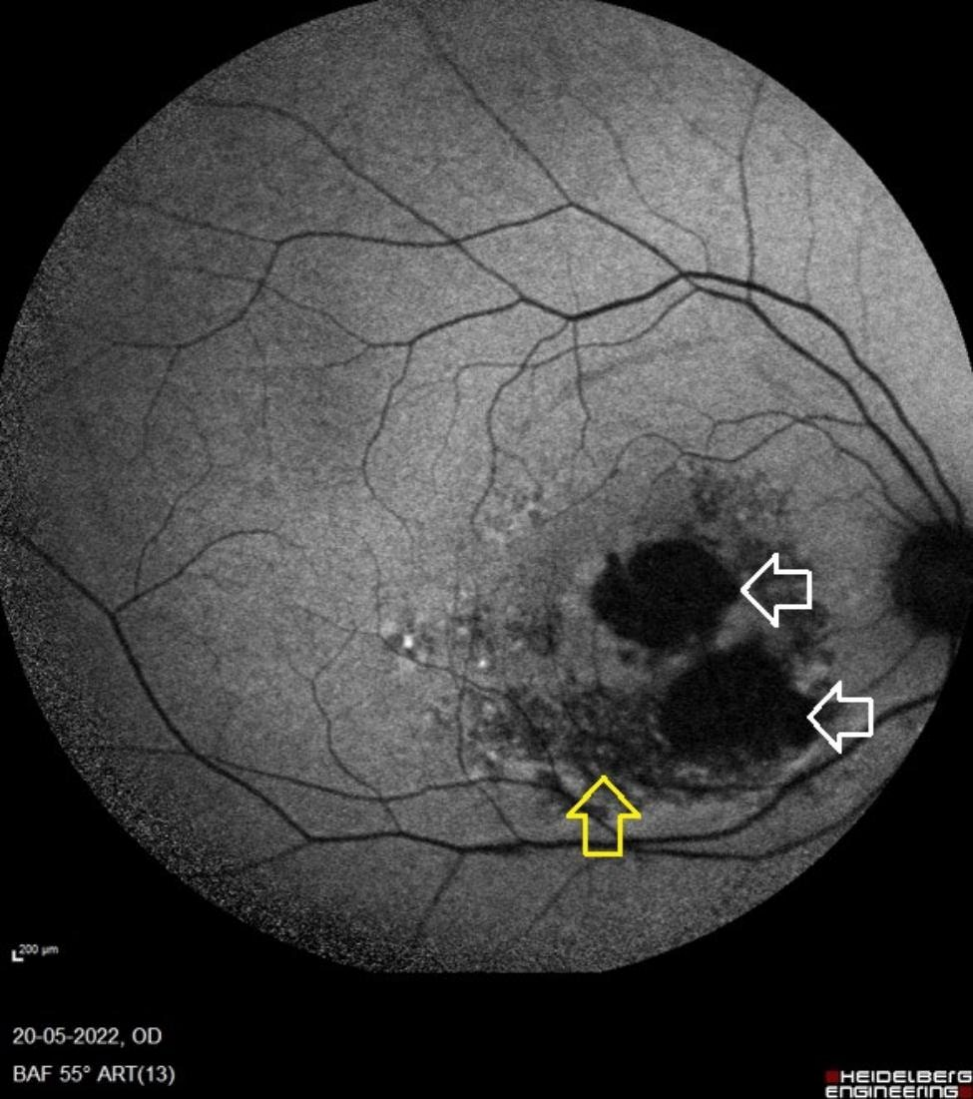
**Blue wavelength fundus autofluorescence image at the 4-year follow-up visit**: The fundus autofluoroscent images shows large areas of hypo autofluorescence suggestive of retinal pigment epithelium (RPE) atrophy. One of these RPE atrophy areas is involving the foveal center (white arrows). Patchy areas of hypoautofluoroscence are noted predominantly inferior to macula suggestive of outer retina and RPE layer damage (yellow arrow)

The patient visited the retina clinic after four years for undergoing cataract surgery on the opposite eye. At this visit, his right eye’s visual acuity remained unchanged at 2/60, N36. Fundus autofluorescence and OCT were performed. The OCT scan revealed a type 1 closure of the macular hole and damage to the outer retinal layers. The RPE layer was absent at the fovea and inferiorly, and the visibility of the underlying choroidal layers was enhanced (Fig. 1G-H). The choroid at the fovea was getting progressively thinner after surgery (Table 1). The enface fundus autofluorescence scan revealed large areas of hypoautofluoroscence suggestive of RPE atrophy, one of which involved the foveal center and measured 4.01 mm^2^ and the other of which was adjacent to the fovea and measured 4.51 mm^2^. On the fundus autofluorescence image, patches of RPE changes were observed (Fig. 2). During this visit, the stage of diabetic retinopathy did not advance. In the previous four years, the patient had no history of any other ocular pathology.

**Table 1:** Choroidal thickness at the fovea at sequential visits:

	Subfoveal choroidal thickness (µm)
Before surgery	339
2-week after surgery	289
6-week after surgery	271
4-year after surgery	257

## Discussion and conclusion

Over a 4-year follow-up period, this case report suggests progressive subfoveal choroidal thinning and damage to the retinal and RPE layers in an eye with combined BBG and endolight-induced retinal toxicity after macular hole repair surgery. In this case, the RPE changes at the macula progressed over time to develop complete RPE atrophy. Such long-term effects of BBG-induced retinal damage have not been described in the literature, to the best of our knowledge.

The macular damage following macular hole repair surgery have been attributed to the combined mechanisms of phototoxicity by the endo illuminator light and BBG dye-induced changes in the outer retina and RPE layers [4, 5]. However, these mechanisms have been responsible for primarily causing the acute changes in the retinal layers. One would expect these changes to be permanent and non-progressive and affect the vision only if the fovea gets involved. In the current case, a continuous change to the outer retinal, RPE and choroidal layers after 4 years following surgery was noted. The possible explanation for this progressive change in the retinal layers and RPE could be as follows: Vital dyes such as BBG and indocyanine green are small chemical molecules that can easily penetrate the retina and remain entrapped within the retinal cells [6]. They produce phototoxic free radicals after absorption of light causing damage to the retinal layers and RPE. The light absorption properties of BBG dye lies between 260 and 900 nm in different solvents [7]. All BBG solutions have double-peak curves. The first peak is noted at 260–280 nm and the second peak occur between 540 and 680 nm. The visible light wavelength ranges from 380 to 700 nm similar to the xenon light wavelength used by the endoilluminator during vitreoretinal surgery [8]. The short wavelength blue light in the visible spectrum gets absorbed by the BBG which produces toxic free radicals and subsequent damage to the retinal layers, photoreceptors and RPE cells and thus causing a continuous damage to the retinal layers. In this case, there was no change in post-operative visual acuity over 4 years because the RPE beneath the fovea was already compromised from the start. However, if the foveal RPE is initially spared from damage, a progression of RPE damage to include the fovea could result in significant visual loss. The presence of damaged photoreceptors and RPE may have resulted in the progressive thinning of the choroidal layers, as the RPE and photoreceptors required less nutrition from the choroid. This explanation may be comparable to that which has been reported for age-related macular degeneration [9].

To summarise, a rare case of continuously progressing RPE layer damage with choroidal thinning over a long follow-up interval was described due to combined BBG-induced dye and endoilluminator toxicity. To avoid any immediate phototoxic damage to the RPE cells and photoreceptors, intra-operative measures such as avoiding repeated ILM with BBG for long periods of time and avoiding use of high focal illumination close to the macula for longer periods of time should be considered. In addition, in patients undergoing vitreous surgery in conjunction with BBG dye use, yellow-tinted intraocular lenses or spectacles may be recommended post-surgery to block the maculotoxic blue light and thus prevent progressive phototoxic damage to the RPE cells.

## Data Availability

The datasets used and/or analysed during the current study are available from the corresponding author on reasonable request.

## Abbreviations

BBG: Brilliant blue G
ILM: Internal limiting membrane
OCT: Optical coherence tomography
RPE: Retinal pigment epithelium
SF6: Sulphur hexafluoride
